# Real-world comparative effectiveness of bDMARDs and JAK inhibitors in elderly patients with rheumatoid arthritis

**DOI:** 10.1097/MD.0000000000031161

**Published:** 2022-10-21

**Authors:** Jumpei Temmoku, Kiyoshi Migita, Shuhei Yoshida, Haruki Matsumoto, Yuya Fujita, Naoki Matsuoka, Makiko Yashiro-Furuya, Tomoyuki Asano, Shuzo Sato, Eiji Suzuki, Hiroshi Watanabe, Masayuki Miyata

**Affiliations:** a Department of Rheumatology, Japanese Red Cross Fukushima Hospital, Yashima, Fukushima, Japan; b Department of Rheumatology, Fukushima Medical University School of Medicine, Hikarigaoka, Fukushima, Fukushima, Japan.

**Keywords:** bDMARDs, cytokine, JAK inhibitors, JAK/STAT pathway, rheumatoid arthritis, tsDMARDs

## Abstract

In this retrospective cohort study, we compared the retention rates and effectiveness of biologic disease modifying antirheumatic drugs (bDMARDs) and targeted synthetic DMARDs (targeted disease modifying antirheumatic drug [tsDMARDs]: Janus kinase inhibitors [JAKi]) in elderly patients with RA. One hundred thirty-four elderly RA patients (≥65 years) who were initiated with bDMARDs (n = 80) or JAKi (n = 54) between 2016 and 2020 in our institute were enrolled in this analysis. Follow-up was conducted at 4-week intervals from the start of bDMARDs or JAKi. We compared the drug retention and clinical response at 24 week between elderly RA patients treated with bDMARDs and JAKi. In the demographic data, more disease duration, the proportion of previous bDMARDs use and less the proportion of glucocorticoid use in JAKi group was significantly observed compared to the bDMARDs group. Otherwise, there was no significant difference in the other variables between the bDMARDs and JAKi groups. In the JAKi group, drug retention rate was not significantly different compared to the bDMARDs group (HR: 0.723, 95% CI: 0.406–1.289, *P* = .266). Also, there was no significant difference in the proportion of patients achieving good or moderate European alliance of associations for rheumatology (EULAR) response at 24 week between these two groups (bDMARDs; 88.6% vs JAKi; 91.8%, *P* = .158). In elderly RA patients initiated with bDMARDs or JAKi, drug retention rates of these targeted therapies did not differ significantly between these two groups. These findings suggest that elderly RA patients can achieve similar clinical improvement after initiating bDMARDs or JAKi.

## 1. Introduction

Rheumatoid arthritis (RA) is an inflammatory disorder characterized by synovitis and progressive joint destruction.^[1]^ Previous studies have demonstrated that elderly RA patients have been shown to be increased and patients with elderly-onset RA (EORA) present with the clinical characteristics that are distinct from those of younger-onset RA (YORA).^[2]^ The presence of multiple comorbidities and the safety concern may influence the treatment selections in elderly patients with RA.^[3]^ Therefore, these therapeutic difficulties may lead to the disability in elderly patients with RA.^[4]^ Furthermore, EORA patients are prone to have more abrupt onset and more aggressive clinical course compared to YORA patients.^[5]^ Despite these severe RA phenotypes, EORA patients tend to be treated with glucocorticoids and lower doses of methotrexate (MTX).^[6]^ Although biologic disease modifying antirheumatic drugs (bDMARDs) are one of the treatment options for elderly patients with RA disease activity, EORA patients are less frequently treated with bDMARDs compared with YORA patients due to the safety concerns.^[7]^ Furthermore, RA patients who are refractory to multiple bDMARDs have been serious issues in clinical practice.^[8]^ Janus kinase inhibitors (JAKi) are the first targeted synthetic disease modifying antirheumatic drugs (tsDMARDs) and be in widespread clinical use used for the treatment of RA, and their efficacy seems to be comparable to those of bDMARDs.^[9]^ In contrast to the single cytokine targeting approach of bDMARDs, JAKi are designed to inhibit signaling through a variety of cytokines implicated in the pathogenesis of RA.^[10]^ Therefore, it is interest to investigate the effectiveness and safety of JAKi in elderly RA patients who are intolerant to conventional synthetic disease modifying antirheumatic drugs (csDMARDs). The safety profile of JAKi has been comparable to that of bDMARD in clinical trial,^[11]^ the real-world data on the effectiveness and safety of JAKi in elderly RA patients are limited. From this viewpoint, we compared the effectiveness and safety of bDMARDs and JAKi in elderly RA patients in this study.

## 2. Methods

### 2.1. Patients and study design

We conducted a retrospective cohort study at the Department of Rheumatology, Fukushima Medical University Hospital and Fukushima Red Cross hospital. Among 600 elderly patients (age ≥65 years; Female 412, Male 188) diagnosed with RA during the study period (between June, 2016 and October 2020), 134 consecutive elderly (age ≥65 years) patients who were initiated with bDMARDs or JAKi were enrolled. All patients were diagnosed as having RA according to the 1987 American College of Rheumatology (ACR) classification criteria for RA^[12]^ and be continuously followed up after initiation of bDMARDs or JAKi. The following bDMARDs were used in our cohort: 18 tumor necrosis factor inhibitors (9 etanercept, 8 golimumab, and 1 certolizumab pegol), 30 interleukin-6 inhibitors (29 tocilizumab or 1 sarilumab), and 32 abatacept. The tsDMARDs included 54 JAKi (14 tofacitinib, 36 baricitinib, and 4 upadacitinib).

### 2.2. Ethics and registration

The study was approved by the Institutional Review Board of Fukushima Medical University (No. 2019-097, December 9th 2021), and Japanese Red Cross Fukushima Hospital (No. 55, February 7th 2022).

### 2.3. Clinical evaluations

At the start of treatment, baseline data were collected from medical records, including demographics, clinical data (disease duration, presence of anticyclic citrullinated protein [CCP] antibody and rheumatoid factor [RF]), evaluations of disease activity (swollen joint count [SJC], tender joint count [TJC], patient global assessment [PtGA], physician global assessment [PGA]), and C-reactive protein [CRP], and information of treatments (current glucocorticoid and MTX doses, previous use of csDMARDs and b/tsDMARDs). Most of the subjected patients received the influenza vaccine that is adjusted each year depending on the strains of influenza predicted for the influenza season. Additionally, most elderly patients (>65 years old) had received at least one Pneumococcal vaccination (23-valent pneumococcal polysaccharide vaccine: PPSV23). However few patients received meningococcal and hepatitis B vaccine. Treatment was at the discretion of the attending physician, based on the clinical conditions and patient’s intentions.

### 2.4. Follow-up

Serial assessments of disease activity including laboratory and treatment-related information were collected at every 4 weeks after the initiation of bDMARDs and JAKi. If treatment was discontinued, the reasons for discontinuations were recorded. Clinical response after 24 weeks from the start of bDMARDs and JAKi was evaluated according to the European alliance of associations for rheumatology (EULAR) response as follows.^[13]^ Good responders were defined as the improvement >1.2, and a present disease activity score 28 (DAS28) ≤3.2. Moderate responders were defined as the improvement >0.6 to ≤1.2, and a present DAS28 ≤5.1; or improvement >1.2, and a present DAS28 >3.2. Nonresponders were defined as the improvement ≤0.6, or improvement >0.6 to ≤1.2, and a present DAS28 >5.1. Adverse events (AEs) that had caused discontinuation of bDMARDs or tsDMARDs were record in detail. Decisions to discontinue of bDMARDs or JAKi due to AEs were determined carefully by the treating physicians based on the evaluation of clinical findings, laboratory data and radiological examinations.

### 2.5. Statistical analysis

Continuous variables were showed as mean ± standard deviation or median (interquartile range) and categorical variables were sowed as frequency (percentage). The chi-squared test was used to compare categorical variables, and the Mann–Whitney *U* test was used to compare continuous variables. Drug retention was analyzed using Kaplan–Meier plots and assessed using the log-rank test. Cumulative incidences of discontinuation due to lack of effectiveness or adverse effects were compared using the Log-rank test for the Kaplan–Meier model. Statistical analyses were performed using the software of SPSS Statistics (version 25.0 for Windows, Chicago, IL). Two–tailed *p* values < .05 were considered indicative of statistical significance.

## 3. Results

### 3.1. Clinical characteristics of elderly RA patients who was initiated with bDMARDs or JAKi

In total 600 elderly RA patients treated in our institute, 134 (22.3%) RA patients (≥65 years) who were initiated with bDMARDs (n = 80) or JAKi (n = 54) were enrolled in this study. Demographic and disease-related characteristics features of the whole elderly RA patients initiated with these targeted therapies are shown in Table 1. The characteristics of patients in each group (bDMARDs vs JAKi) are shown in Table 2. The number of patients initiated with JAKi was relatively small. Two different patient groups (bDMARDs and JAKi) had divergent baseline disease characteristics, more disease duration, the proportion of previous bDMARDs use and less the proportion of glucocorticoid use in JAKi group was significantly observed compared to these in bDMARDs group. There was no significant between-group difference with respect the other variables.

**Table 1:** Baseline characteristics of elderly RA patients at initiation of bDMARD or JAKi.

Characteristic	n = 134
Age (yr), median (IQR)	74 (69–80)
Female, n (%)	99 (73.9%)
Disease duration (yr), median (IQR)	8.8 (2.4–17.1)
RF-positive, n (%)	102 (76.1%)
ACPA-positive, n (%)	88 (65.7%)
CRP (mg/dL), median (IQR)	1.45 (0.49–3.28)
DAS28-CRP, median (IQR)	4.2 (3.4–5.0)
eGFR (mL/min), median (IQR)	77 (60.1–104)
Interstitial lung disease, n (%)	22 (16.4%)
MTX use, n (%)	61 (45.5%)
MTX dose (mg/wk), median (IQR)	6 (4–8)
GC use, n (%)	48 (35.8%)
GC dose (mg/day), median (IQR)	5 (3–7)
Other csDMARDs use, n (%)	41 (30.6%)
Prior bDMARDs use, n (%)	49 (36.6%)

ACPA = anti-citrullinated protein antibody, bDMARDs = biological disease-modifying anti-rheumatic drugs, CRP = c-reactive protein, csDMARDs = conventional synthetic disease-modifying anti-rheumatic drugs, DAS28-CRP = disease activity score28-c-reactive protein, eGFR = estimated glomerular filtration rate, GC = glucocorticoid, IQR = interquartile range, JAKi = Janus kinase inhibitors, MTX = methotrexate, RA = rheumatoid arthritis, RF = rheumatoid factor.

**Table 2:** Comparison of characteristics between bDMARDs group and JAKi group in elderly RA patients.

	bDMARDs (n = 80)	JAKi (n = 54)	*p* value
Age (yr), median (IQR)	73 (68–79)	75 (70–81.3)	.08
Female, n (%)	60 (75%)	39 (72.2%)	.72
Disease duration (yr), median (IQR)	7 (1.4–14.8)	11 (3.4–22.3)	.049*
RF-positive, n (%)	64 (81%)	38 (76%)	.495
ACPA-positive, n (%)	54 (73.9%)	34 (79%)	.536
CRP (mg/dL), median (IQR)	1.37 (0.31–3.8)	1.47 (0.58–3.04)	.896
DAS28-CRP, median (IQR)	4.01 (3.3–4.81)	4.44 (3.67–5.03)	.092
eGFR (mL/min), median (IQR)	71 (53.3–83)	72.3 (59.2–84.3)	.476
Interstitial lung disease, n (%)	13 (16.3%)	9 (16.7%)	.949
MTX use, n (%)	37 (46.3%)	24 (44.4%)	.837
MTX dose (mg/wk), median (IQR)	6 (6–8)	6 (4–8)	.688
GC use, n (%)	39 (48.8%)	9 (16.7%)	<.001*
GC dose (mg/d), median (IQR)	5 (3–7.5)	3.5 (1–5.5)	.201
Other csDMARDs use, n (%)	35 (43.8%)	6 (11.1%)	<.001*
Prior bDMARDs use, n (%)	22 (27.5%)	27 (50%)	.008*
Follow up periods (mo), median (IQR)	20 (13–29.8)	24.5 (12–35.5)	.26

ACPA = anti-citrullinated protein antibody, bDMARDs = biological disease-modifying anti-rheumatic drugs, CRP = c-reactive protein, csDMARDs = conventional synthetic disease-modifying anti-rheumatic drugs, DAS28-CRP = disease activity score28-c-reactive protein, eGFR = estimated glomerular filtration rate, GC = glucocorticoid, IQR = interquartile range, JAKi = Janus kinase inhibitors, MTX = methotrexate, RA = rheumatoid arthritis, RF = rheumatoid factor.

* *P* < .05.

### 3.2. Drug retention rates and reasons for discontinuation

**Figure 1.**
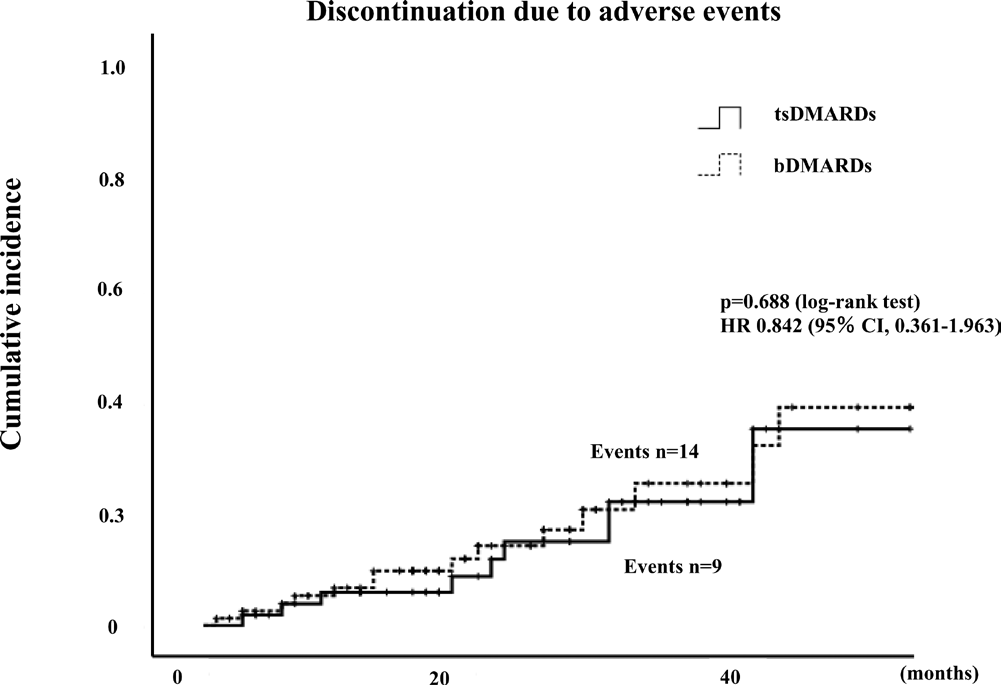
Cumulative incidences of discontinuation of bDMARDs and JAKi due to adverse. In the JAKi group, the incidence of drug discontinuation due to adverse effects was not significantly different compared to the bDMARDs group (HR: 0.842, 95% CI: 0.361–1.963, *P* = .688). bDMARDs = biological disease-modifying anti-rheumatic drugs.

**Figure 2.**
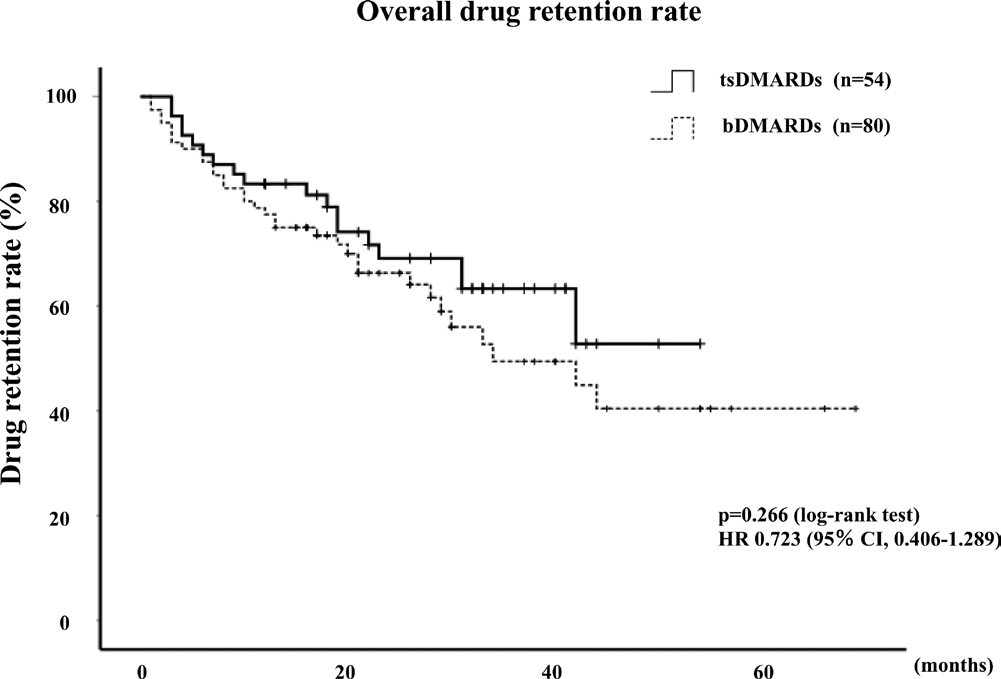
Kaplan–Meier curve related to the overall cumulative drug retention rate of bDMARDs and JAKi in elderly patients initiating these molecular targeting treatment. In the JAKi group, drug retention rate was not significantly different compared to the bDMARDs group (HR: 0.723, 95% CI: 0.406–1.289, *P* = .266). bDMARDs = biological disease-modifying anti-rheumatic drugs.

**Figure 3.**
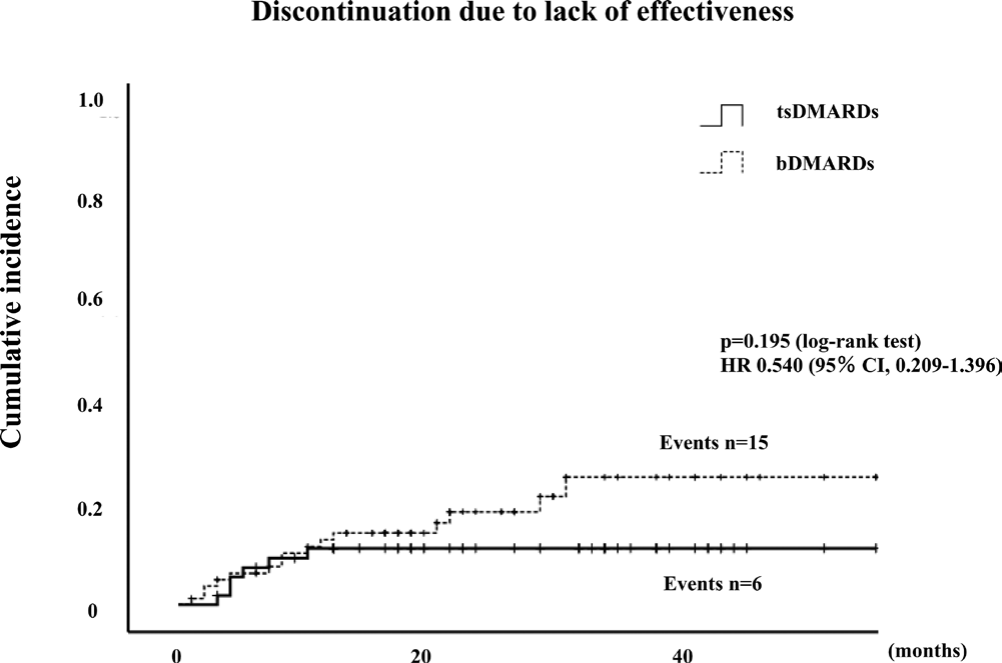
Cumulative incidences of discontinuation of bDMARDs and JAKi due to lack of effectiveness. In the JAKi group, the incidence of drug discontinuation due to lack of effectiveness was not significantly different compared to the bDMARDs group (HR: 0.540, 95% CI: 0.209–1.396, *P* = .195). bDMARDs = biological disease-modifying anti-rheumatic drugs.

Among 80 patients initiated with bDMARDs, treatment was discontinued in 15 patient (18.8%) due to insufficient effectiveness, 14 patients (17.5%) due to AEs, including impairment of infection (3; 21.4%), neoplasms (3; 21.4%), cardiovascular complications (2; 14.3%), allergic reaction (2; 14.3%), impairment of liver function (1; 7.1%), renal dysfunction (1; 7.1%), hematological complications (1; 7.1%), hypothyroidism (1; 7.1%) and 2 patients due to remission, 1 patient due to patient preference. Among 54 patients initiated with JAKi, treatment was discontinued in 6 patients (11.1%) due to insufficient effectiveness, 8 patients (14.8%) due to AEs, including neoplasms (2; 25%), gastrointestinal complications (2; 25%), cardiovascular complications (1; 12.5%), infection (1; 12.5%), impairment of liver function (1; 12.5%), hematological complications (1; 12.5%). None of the patients in either group experienced any life-threatening AEs. In the JAKi group, the incidence of drug discontinuation due to adverse effects was not significantly different compared to the bDMARDs group (HR: 0.842, 95% CI: 0.361–1.963, *P* = .688) (Fig. 1). The overall drug retention rates of bDMARDs and JAKi are shown in Figure 2. The retention rate of JAKi group was not significantly different compared to these bDMARDs group (HR: 0.723, 95% CI: 0.406–1.289, *P* = .266). Cumulative incidence of drug discontinuation due to lack of effectiveness were also compared between these two groups. The incidence of drug discontinuation due to lack of effectiveness in the JAKi group was not significantly different compared to the bDMARDs group (HR: 0.540, 95% CI: 0.209–1.396, *P* = .195) (Fig. 3).

### 3.3. Drug effectiveness at 24 weeks follow-up

**Figure 4.**
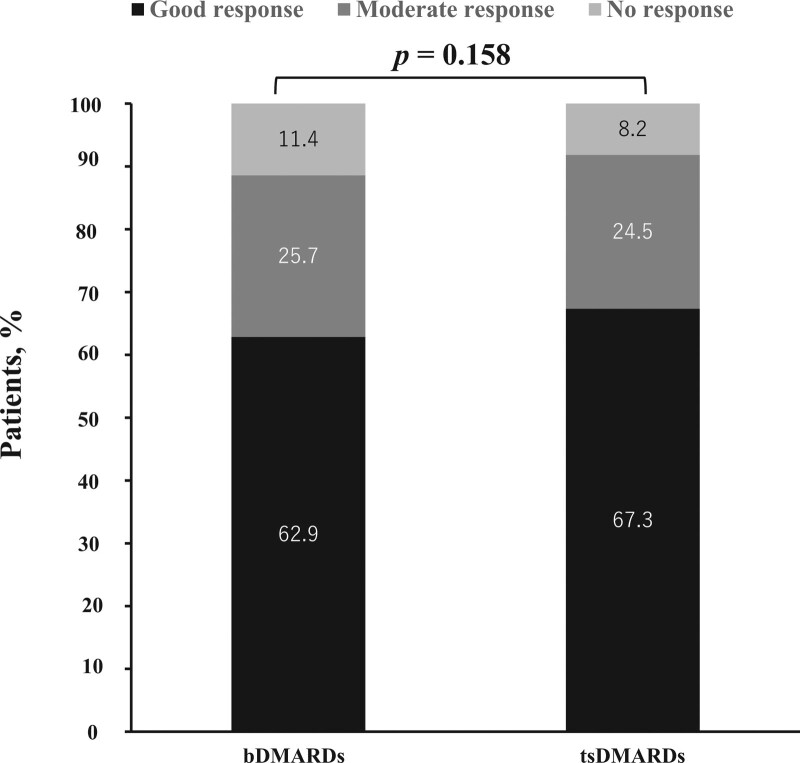
Proportion of patients achieving EULAR response between elderly patients initiating with bDMARDs and JAKi at 24 week. There was no significant difference between elderly patients initiating bDMARDs and JAKi groups (bDMARDs; 88.6% vs JAKi; 91.8%, *P* = .158). bDMARDs = biological disease-modifying anti-rheumatic drugs, EULAR = European alliance of associations for rheumatology, JAKi = Janus kinase inhibitors.

Clinical response according to EULAR response after 24 weeks were compared bDMARDs and JAKi groups (Fig. 4). At week 24, the proportion of patients achieving good/moderate EULAR response seems to be higher in JAKi group, however there was no significant difference between elderly patients initiating bDMARDs and JAKi groups (bDMARDs; 88.6% vs JAKi; 91.8%, *P* = .158).

## 4. Discussion

Elderly patients with RA had more comorbidities which may lead to the functional disability.^[14]^ Additionally, these elderly patients are prone to be intolerant to MTX or other csDMARDs.^[15]^ In general, bDMARDs were shown the similar treatment response between EORA and YORA.^[16]^ The efficacy of JAKi seems to be comparable in elderly RA patients compared to younger RA patients in clinical trial.^[17]^ In the RA-BUILD and RA-BEAM studies, baricitinib showed similar efficacy between young (<50 years) and old (≥65 years) patients.^[17]^ However, elderly patients are likely to experience more AEs under the treatment of the JAKi, tofacitinib,^[18]^ and the safety of JAKi in elderly patients may not be completely elucidated. Clinical trial data demonstrated that JAKi showed the clinical improvements in RA patients with MTX-inadequate response (MTX-IR). In head-to-head trials against TNFi, baricitinib showed the higher clinical responses compared to those of adalimumab.^[19]^ However, the effectiveness or safety of JAKi in elderly RA patients is yet to be evaluated in real-world elderly RA patients. In this observational study subjected elderly (aged ≥65 years) RA patients, we compared the effectiveness and retention rates of bDMARDs and JAKi. We found no significant difference in the overall drug retention rates between bDMARDs and JAKi. To the best to our knowledge, this is the first study to compare the drug retention rates and safety of bDMARD and JAKi in elderly RA patients with moderate to high disease activity. In particular, we report on the real-world experience of elderly RA patients treated with JAKi. The rates of elderly RA patients achieving good/moderate EULAR response at 24 weeks were comparable in the bDMARDs and JAKi groups. Our results suggest that the effectiveness and safety of JAKi are comparable to those of bDMARDs even in elderly RA patients. RA is characterized by the overproduction of inflammatory cytokines which use the Janus kinase/signal transducer and activator of transcription (JAK/STAT) pathways in the receptor signaling.^[20]^ Therefore, JAKi may exert their effectiveness by targeting the multiple cytokines cascades.^[10,21]^ Although, the difference in the efficacy and safety of these JAKi according to their JAK-isoform-selectivity remains to be elucidated,^[22]^ the efficacy appears to be comparable to that of bDMARDs.^[23]^ Given that the drug retention may depend on effectiveness and safety, the comparison of drug retention rate may reflect the efficacy and safety of bDMARDs or JAKi in elderly patients with RA. A systematic review revealed similar outcomes of JAKi and bDMARDs therapy.^[24]^ Consistent with these reports, in our cohort, the effectiveness of JAKi was comparable to that of bDMARDs. The incidence of drug discontinuation due to lack of effectiveness was comparable in the two groups. Therefore, our data suggest that JAKi is a potential therapeutic option for elderly patients who are refractory to csDMARDs. However, it does not mean that JAKi should be recommended to most elderly RA patient with high disease activity. Since elderly RA patients are frequently accompanied with a variety of comorbidities which may affect the treatment choice of JAKi.^[25]^ Therefore, an appropriate choice of JAKi should be determined after the careful consideration of comorbidities, and the balance of risk and benefit in elderly RA patients. Further large prospective studies may draw the more detailed evidence for the treatment decision in elderly RA patients.

The limitations of our study were as follows. First, this was a retrospective observational study which may affect the evaluation of treatment effectiveness. Second, the sample size was relatively small. Third, the assessment of RA disease activity was performed using DAS28-CRP. Finally, TNF inhibitors, IL-6 receptor antibodies and abatacept were analyzed collectively as bDMARDs and the characteristics of each bDMARDs may not have been reflected. Similarly, tofacitinib, baricitinib and upadacitinib were analyzed collectively as JAKi and the characteristics of each JAKi may not have been reflected.

## 5. Conclusions

We compared the drug retention rates and effectiveness of bDMARDs and JAKi in elderly RA patients. There was no significant difference in the overall drug retention rates between bDMARDs and JAKi. Additionally, elderly RA patients initiated with bDMARDs or JAKi showed similar treatment effectiveness. Further investigations using larger sample size are needed to draw the evidence on the effectiveness and safety of JAKi in elderly RA patients.

## Acknowledgements

We are grateful to Ms Sachiyo Kanno for her technical assistance in this study.

## Author contributions

**Conceptualization:** Kiyoshi Migita.

**Data curation:** Jumpei Temmoku, Shuhei Yoshida, Haruki Matsumoto, Yuya Fujita, Naoki Matsuoka, Makiko Yashiro-Furuya, Tomoyuki Asano, Shuzo Sato, Eiji Suzuki, Hiroshi Watanabe.

**Formal analysis:** Jumpei Temmoku.

**Funding acquisition:** Jumpei Temmoku.

**Software:** Jumpei Temmoku.

**Supervision:** Masayuki Miyata.

**Writing – original draft:** Jumpei Temmoku, Kiyoshi Migita.

**Writing – review & editing:** Jumpei Temmoku, Kiyoshi Migita.
